# The relationship between vitamin D supplementation and mortality in critically ill patients

**DOI:** 10.1186/2197-425X-3-S1-A362

**Published:** 2015-10-01

**Authors:** HK Atalan, B Gucyetmez, T Sarikayo, UA Turan, E Ozden, M Berktas, N Cakar

**Affiliations:** Anesthesiology and Reanimation, Atasehir Memorial Hospital, Istanbul, Turkey; İntensive Care, Acibadem International Hospital, İstanbul, Turkey; İntensive Care, Acibadem Kozyatagi Hospital, İstanbul, Turkey; İntensive Care, Acibadem Kadikoy Hospital, İstanbul, Turkey; İntensive Care, Antalya Memorial Hospital, Antalya, Turkey; PEPIRC, Yeditepe University, İstanbul, Turkey; Anesthesiology and İntensive Care, Acibadem University Medical Faculty, İstanbul, Turkey

## Introduction

Vitamin D is a fat soluble vitamin that play a major role in the regulation of bone metabolism, and has effects on immun, cardiac and vascular systems [1]. Vitamin D deficiency is common in the general population as well as the critically ill patients and was reported to be associated with increased mortality and morbidity [2, 3].

## Objectives

To evaluate relationship between vitamin D supplementation at the ICU admission and mortality.

## Methods

Upon the approval of local ethical committee, a total of 491 patients admitted to ICU of two centers between January 2014 and January 2015 were evaluated retrospectively. The patients who were under 18 years old, elective surgery and whose serum vitamin D levels and outcomes were unknown were excluded from the study. A total of 213 patients were included in the study and divided into 2 groups in accordance with their vitamin D serum level as low Vit-D group ( < 25ng/dL) and normal Vit-D group (≥25ng/dL). Patients with low serum vitamin D level received vitamin D orally or via nasogastric tube once at a dose of 600000 iu. The patient's age, gender, APACHE II scores, number of failed organ systems, serum vitamin D levels, length of ICU stay and mortality were recorded. Groups were compared by using Mann Whitney U test due to non-normal distribution pattern.

## Results

Low Vit-D group was made up of 166 (78%) patients and normal Vit-D group was made up of 47 (22%) patients. Groups were similar in terms of age (61 vs 63), gender (male, 65% vs 68%), APACHE II score (21 vs 20), number failed organ systems (1 vs 1), length of ICU stay (12 vs 10) and the ICU mortality (22.9% vs 17%) (p > 0.05 for each).

## Conclusions

Vitamin D deficiency was found to be frequent at the ICU admission in our study. Mortality rates were similar in both groups, therefore vitamin D supplementation may have beneficial effects on mortality in critically ill patients with low serum vitamin D levels in ICU admission.
